# mTOR inhibition reduces cellular proliferation and sensitizes pituitary adenoma cells to ionizing radiation

**DOI:** 10.4103/2152-7806.77029

**Published:** 2011-02-23

**Authors:** Sangeetha Sukumari-Ramesh, Nagendra Singh, Krishnan M. Dhandapani, John R. Vender

**Affiliations:** Department of Neurosurgery, Medical College of Georgia, Augusta, GA 30912, USA; 1 Department of Biochemistry, Medical College of Georgia, Augusta, GA 30912, USA

**Keywords:** Akt, everolimus, mTOR, pituitary tumor, radiation, rapamycin, RAD001

## Abstract

**Background:**

Pituitary adenomas are the most frequent brain tumor in adults. Although histologically benign, pituitary tumors cause significant morbidity and mortality. Neurosurgery and medical therapeutics may lessen the morbidity and mortality associated with pituitary tumors; however, these treatments are associated with significant adverse side effects. Thus, an improved understanding of pituitary adenomas at the molecular and cellular level is needed to design novel therapeutic compounds.

**Methods:**

To assess the effect of mammalian target of rapamycin (mTOR) inhibition on pituitary adenoma cells, rat GH3 or MMQ cells were treated with the clinically useful mTOR inhibitors, rapamycin or RAD001. Cellular proliferation and growth following exposure to mTOR inhibitors or radiation were assessed using biochemical methods.

**Results:**

In the present study, we observed basal activation of mTOR, downstream of constitutive Akt signaling, in rat GH3 adenoma cells. Functionally, the mTOR inhibitors, rapamycin and RAD001 (500 pM–5 nM), induced G1 growth arrest within 24 hours, an effect associated with reduced cellular proliferation. Both rapamycin and RAD001 decreased the phosphorylation of mTOR at the serine 2448, a key determinant of mTOR activity. Inhibition of mTOR also radiosensitized GH3 cells such that 2.5 Gy in combination with 500 pM rapamycin or RAD001 reduced cellular viability more effectively than 2.5 or 10 Gy alone.

**Conclusions:**

These data may support a possible therapeutic role for mTOR inhibitors in limiting the cellular proliferation and radioresistance of pituitary adenoma cells.

## INTRODUCTION

Pituitary tumors are among the most prevalent brain tumors in adults and may be associated with significant morbidity and mortality. Although histological malignancy of the pituitary is rare, adenomas may exhibit infiltrative and invasive growth patterns characteristic of malignant tumors.[21] Surgical resection and medical therapies improve patient prognosis; however, lifelong palliative treatment options are associated with a poor compliance rate and numerous adverse side effects.[20,21] Similarly, gamma knife radiosurgery is limited by the sensitivity of the optic chiasm to radiation damage at doses exceeding 8 Gy.[10] Thus, the identification of novel therapeutic targets to limit cellular growth and/or to enhance radiosensitivity of pituitary adenoma may significantly improve patient care.

Akt (protein kinase B) is a cellular serine/threonine protein kinase that has been implicated in cellular growth and survival.[1] Akt expression and activity were constitutively increased in human pituitary tumors, as compared to normal pituitary tissue,[14] suggesting a possible role for Akt in the pathogenesis of pituitary adenomas. In support of this notion, our laboratory first reported that the constitutive activation of Akt promoted the growth and survival of GH3 pituitary adenoma cells;[22] however, the downstream mechanisms underlying this effect remain unexplored and could provide novel opportunities for therapeutic intervention.

Mammalian target of rapamycin (mTOR), a serine/threonine kinase involved in diverse cellular processes, including protein translation, mRNA turnover, and protein stability, mediates, at least in part, some of the biological actions of Akt.[2,3,11] Based on our recent demonstration that basal Akt activation increased cellular growth in pituitary adenoma, we herein tested the hypothesis that mTOR activation, downstream from Akt signaling, may increase the growth and survival of pituitary adenoma cells. Our data suggest mTOR promotes pituitary adenoma growth and radioresistance, indicating a possible therapeutic role for mTOR inhibitors in the clinical management of pituitary tumors.

## MATERIALS AND METHODS

### Supplies

All cell culture reagents, sera, and media were purchased from Hyclone Laboratories (Logan, UT, USA). The mTOR inhibitors, rapamycin and RAD001 (everolimus), were purchased from Alexis Biochemicals (San Diego, CA, USA) and Sigma (St. Louis, MO, USA), respectively.

### Cell culture

Rat GH3 and mouse MMQ pituitary adenoma cells (American Type Tissue Collection, Manassas, VA, USA) were cultured in Dulbecco’s modified Eagle’s medium (DMEM) supplemented with 5% fetal bovine serum, 5% bovine growth serum, and antibiotics in a 37°C humidified incubator at 5% CO_2_. GH3 cells are a well-characterized model of secretory adenomas that hypersecrete both prolactin (PRL) and growth hormone (GH) and that reflect the biology of human secretory tumors.[5–7,16]

### Cellular viability assays

Cell viability was estimated by the addition of 5 mg/mL 3-(4,5-dimethylthiazol-2-yl)-2,5-diphenyltetrazolium bromide (MTT) followed by incubation for 4 hours at 37°C, as described by our laboratory.[22] MTT is a yellow substrate that is only cleaved by active mitochondria in living cells to yield a blue formazan product. Following incubation, formazan crystals were solubilized in 0.04 M hydrochloric acid in isopropanol and absorbance was detected at 540 nm. Viability was normalized to the control, which was considered to represent 100%. Cell death was quantified by measuring the release of the cytosolic enzyme, lactate dehydrogenase (LDH), into the media by damaged or dying cells, as described by our laboratory.[22] LDH release produces a red formazan product, which was detected at an absorbance of 490 nm. Samples were assayed in duplicate and background LDH levels (media alone) were subtracted from all samples. Values are expressed as % LDH released (LDH release/total LDH in the culture), determined following exposure to 2% Triton X-100.

### Cell cycle analysis

5 × 10^4^ cells were plated in a 60-mm plate for 48 hours. Exponentially growing cells were synchronized in serum-free DMEM overnight, and then cells were treated for up to 72 hours in complete culture media containing rapamycin or RAD001. Cells were washed twice, and then permeabilized with 70% methanol overnight at −20°C. Cells were incubated with 0.5 mL of a 50 *μ*g/mL propidium iodide (PI) solution containing 200 *μ*g/mL RNaseA for 30 minutes, and the cells were immediately analyzed by flow cytometry using BD Biosciences FACS Calibur analyzer.

### Western blotting

Following treatments, the cells were washed with phosphate-buffered saline (PBS) and whole cell lysates were collected in radioimmunoprecipitation (RIPA) buffer containing protease inhibitor cocktail, phosphatase inhibitor cocktail and phenyl methane sulfonyl fluoride (PMSF). Following sonication, cell lysates were centrifuged for 5 minutes at 14,000 rpm at 4°C, and protein concentrations were quantified by BCA protein assay kit (Pierce, Rockford, IL, USA). 30 *μ*g of protein were resolved on 4–20% sodium dodecyl sulfate-polyacrylamide gel and transferred onto a polyvinylidene difluoride (PVDF) membrane. Blots were incubated overnight at 4°C in primary antibody (1:500 anti-mTOR antibody or 1:500 anti-phospho-mTOR) (Cell Signaling Technology, Beverly, MA, USA) followed by a 2 hour incubation with an AlexaFluor 750 secondary antibody at room temperature. Blots were visualized using the Li-Cor Odyssey near-infrared imaging system and densitometry analysis was performed using Quantity One software (Bio-Rad, Foster City, CA, USA).

### Radiation studies

3 × 10^4^ cells/well were plated in a 24-well plate for 48 hours. Exponentially growing cells were pretreated with rapamycin or RAD001 for 3 hours and then irradiated using a Nordion Gammacell 40 Exactor Irradiator. Following irradiation, the media were replaced with complete culture media and cells incubated for 48 hours at 37°C, prior to the determination of cellular viability.

## RESULTS

### mTOR inhibition induces G1 growth arrest in pituitary adenoma cells

**Figure 1:**
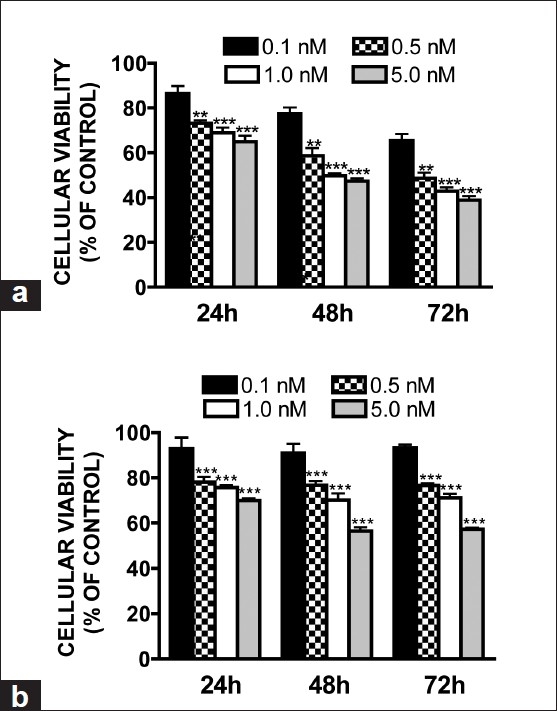
Effect of rapamycin or RAD001 on cellular viability. (a) Rapamycin or (b) RAD001 (0.1–5 nM) reduced cellular viability in GH3 pituitary adenoma cells in a time- and concentration-dependent manner. Cellular viability was assessed at 24, 48, or 72 hours following treatment, and data were normalized to the vehicle-treated group. Data were compared by one-way ANOVA followed by Dunnett’s post-hoc test (**P < 0.01, ***P < 0.001 vs. vehicle-treated cultures)

The mTOR inhibitors, rapamycin and RAD001, reduced the cellular viability of GH3 [Figure 1a] and MMQ (data not shown) cells in a time- and concentration-dependent manner. Rapamycin (500 pM) reduced cellular growth by 27, 41, and 51% at 24, 48, and 72 hours, respectively (*P* < 0.01 vs. vehicle at all timepoints). A maximal response was observed with 1 nM rapamycin, which elicited a 57% reduction in cell viability following a 72 hour treatment (*P* < 0.001 vs. vehicle, not significantly different from 5 nM). Similarly, RAD001 (500 pM) reduced cellular growth by 22, 24, and 24% at 24, 48, and 72 hours, respectively (*P* < 0.001 vs. vehicle at all timepoints) with a maximal effect (44% reduction in viability) observed following a 48 hour treatment with 5 nM (*P* < 0.001 vs. vehicle; *P* < 0.01 vs. 1 nM) [Figure 1b]. In contrast to rapamycin, the anti-proliferative effect of RAD001 was restricted to the first 48 hours of treatment, as cellular viability at the 72 hour timepoint was not significantly decreased as compared to the 48 hour time point. Neither rapamycin nor RAD001 increased cellular LDH release, a measure of cell death, at any timepoint (data not shown), which is indicative of a possible cytostatic effect of both drugs.

**Figure 2:**
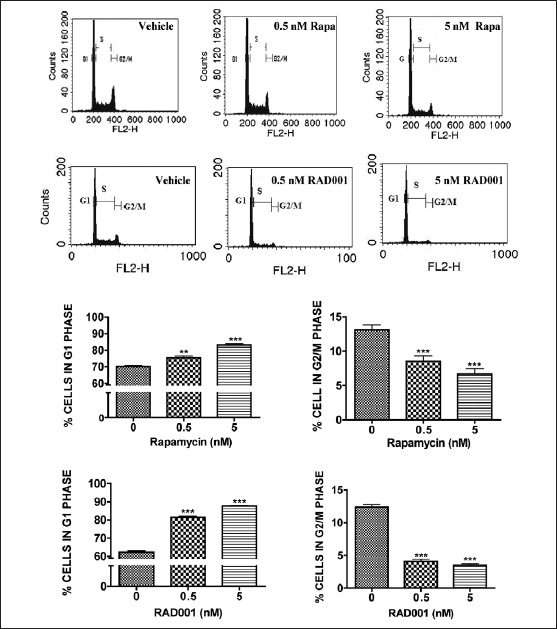
mTOR inhibition induces G1 growth arrest. GH3 cells were treated with rapamycin (0, 0.5, or 5 nM; top panels) or RAD001 (0, 0.5, or 5 nM; bottom panels) for 72 hours and then analyzed using flow cytometry. Both the compounds increased the percentage of cells in G1 phase and decreased the percentage of cells in G2/M phase of the cell cycle. Data were analyzed using one-way ANOVA followed by Dunnett’s post-hoc test (**P < 0.01 and ***P < 0.001 vs. vehicle-treated cultures)

Inhibition of the mTOR pathway appeared to influence cellular proliferation; thus, the effect of rapamycin and RAD001 on cell cycle progression was investigated. Rapamycin (500 pM or 5 nM) significantly increased the percentage of cells in the G1 phase [75.4 ± 1.2%, 83.3 ± 0.8% respectively vs control 70.17± 0.7530], with a concomitant decrease in the percentage of cells within the G2/M phase (8.5 ± 0.8%, 6.7 ± 0.7%, respectively vs. control, 13.1 ± 0.8%) [Figure 2]. Similarly, RAD001 induced G1 growth arrest within 24 hours [Figure 2]; however, unlike rapamycin, the cytostatic effect of RAD001 was restricted to the first 72 hours of treatment (data not shown). Consistent with the lack of LDH release, neither rapamycin nor RAD001 increased the percentage of cells within the sub-G1 fraction, a measure of cell death.

### Rapamycin and RAD001 reduce mTOR activity

**Figure 3:**
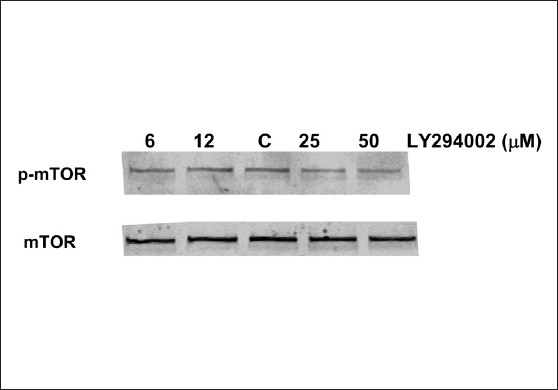
LY294002 attenuates basal activation of mTOR. 18-hour treatment with the PI3K inhibitor, LY294002 (6–50 μM) reduced mTOR phosphorylation on serine 2448, an important residue involved in mTOR activity, as compared to control (C). LY294002 did not affect total mTOR protein

**Figure 4:**
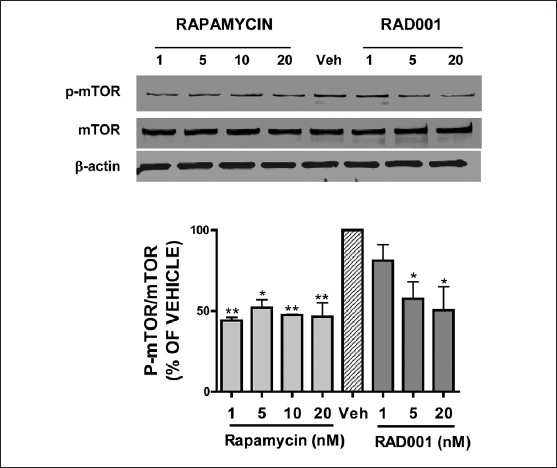
Rapamycin and RAD001 reduce mTOR activity. Treatment with rapamycin (1–20 nM) or RAD001 (1–20 nM) for 24 hours reduced mTOR phosphorylation on serine 2448, an important residue involved in mTOR activity (top panel). Neither rapamycin nor RAD001 affected total mTOR protein. β -actin was used as a loading control. Densitometric analysis of phospho-mTOR/mTOR is depicted in the bottom panel. Data were analyzed using one-way ANOVA followed by Dunnett’s post-hoc test (*P < 0.05 and **P < 0.01 vs. vehicle-treated cultures)

Phosphorylation of serine 2448, a key determinant of mTOR activity, was basally increased in GH3 adenoma cells. Consistent with a role for Akt in the constitutive activation of mTOR, phosphorylation of serine 2448 was reduced by treatment with LY294002, an Akt pathway inhibitor [Figure 3]. Similarly, both rapamycin and RAD001, at concentrations which reduced cellular proliferation, attenuated the phosphorylation of mTOR without influencing mTOR protein expression [Figure 4]. Rapamycin (1, 5, 10, 20 nM) maximally reduced mTOR activation to 44.0, 52.0, 47.5, 46.5% of control, respectively, within 24 hours, whereas RAD001 (1, 5, and 20 nM) decreased mTOR phosphorylation to 81, 57, and 50% of control, respectively.

### mTOR inhibition reduces radioresistance in GH3 adenoma cells

**Figure 5:**
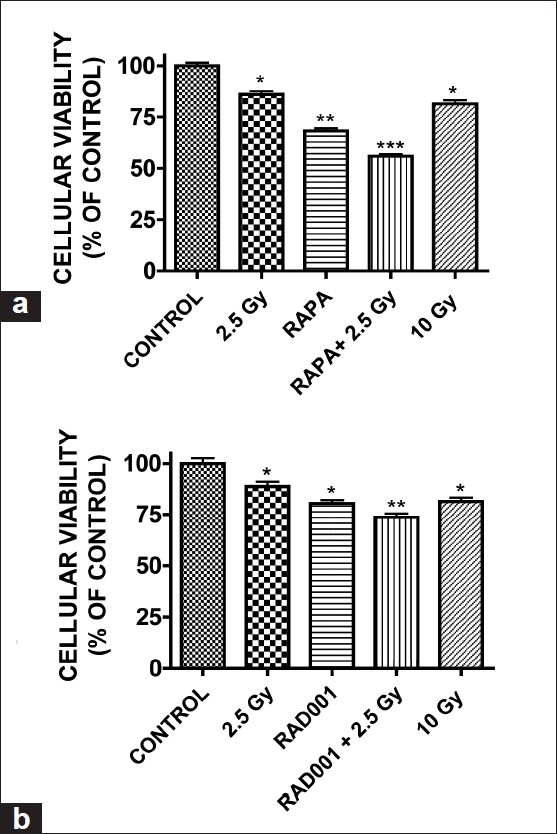
mTOR inhibition radiosensitizes GH3 adenoma cells. Cells were pretreated with (a) rapamycin (0.5 nM) or (b) RAD001 (0.5 nM) for 3 hours and then irradiated (2.5 or 10 Gy). Cell viability was assessed 48 hours following irradiation. Data were analyzed using one-way ANOVA followed by Dunnett’s *post-hoc* test (*P < 0.05, **P < 0.01, ***P < 0.001 vs. vehicle-treated cultures)

G1 growth arrest may increase cellular sensitivity to radiation; thus, the effect of mTOR inhibition on radiation-induced cell death was next assessed. 2.5 and 10 Gy alone reduced cellular viability by 15 and 19%, respectively. In contrast, cellular viability was reduced by 45 and 27% when 2.5 Gy was combined with a 3h pretreatment with either rapamycin (0.5 nM) or RAD001 (0.5 nM), respectively [Figure 5a and 5b]. The radiosensitizing effects were partly attributed to the abrogation of radiation induced G2/M arr and induction of apoptosis as assessed by flow cytometry (unpublished observation).

## DISCUSSION

Aberrant activation of mTOR, an important serine/threonine protein kinase involved in protein translation, cellular proliferation, cellular motility, and cellular survival,[11] promotes the growth of malignant tumors such as glioblastoma multiforme;[17] however, a role for this pathway in the proliferation and survival of benign pituitary adenoma cells remained unknown. Clinically achievable concentration of the mTOR inhibitors, rapamycin and RAD001, reduced cellular proliferation and radiosenstized pituitary adenoma cells, at least in part, by inducing G1 growth arrest. Together, these data suggest, for the first time, a potential role for mTOR inhibitors in the treatment of pituitary tumors, both as a cytostatic agent and as an adjunct to radiation.

Pituitary adenoma accounts for approximately 15% of primary intracranial tumors.[13] Although histologically benign, pituitary tumors cause significant morbidity and mortality due in part to hypersecretion of peptide hormones and to a mass effect caused by cellular proliferation (e.g. compression of the optic apparatus). In the case of secretory tumors, current medical therapies may limit hormone hypersecretion; however, these therapies require lifelong use and may lead to patient non-compliance due to associated adverse side effects.[20] Endocrinologically active pituitary adenomas also necessitate high-dose radiotherapy to limit tumor progression, although long-term risks include hypopituitarism, visual changes and damage to the optic chiasm, and radiation necrosis.[8,9] The limitations in the current clinical management of pituitary adenoma emphasize the need for novel therapeutic targets and improved drug design, which may improve patient outcome.

Increased expression and activity of the serine/threonine kinase, Akt, was observed in human pituitary adenomas, as compared to the normal pituitary gland; however, the functional significance of Akt activation in the progression of pituitary adenoma remains unknown. Similarly, the downstream effectors of Akt signaling remain poorly defined within pituitary adenoma. Recent work done in our laboratory found that Akt was constitutively activated in GH3 adenoma cells, as was observed in human pituitary tumors, and that attenuation of this signaling pathway significantly reduced cellular viability. The effect of Akt was partially mediated by the NFkB transcription factor, although other signaling pathways also contributed to the effect on cellular growth.[22] In the present report, inhibition of Akt reduced the basal activity of mTOR, suggesting mTOR may be an important downstream mediator of Akt.

Basal mTOR functionally contributed toward the cellular growth of pituitary adenoma cells as pharmacological inhibition of mTOR limited cellular proliferation. Unfortunately, rapamycin is highly lipophilic and exhibits poor stability in vivo. These properties potentially limit the clinical usefulness of rapamycin in the management of pituitary tumors; however, more stable and soluble rapamycin analogues, such as RAD001, may retain biological activity against mTOR, and thus, could be more amendable to clinical use. In support of this possibility, oral administration of RAD001 was approved by the Food and Drug Administration (FDA) in early 2009 for the treatment of advanced renal carcinoma. In the present study, both rapamycin and RAD001 attenuated mTOR activation and increased acute G1 growth arrest in pituitary adenoma cell lines. In GH3 cells, 410 pM rapamycin was the IC50 concentration, whereas 986 pM RAD001 was required to exert a similar effect. Similarly, 4.7 *μ*M rapamycin and 8.6 *μ*M RAD001 were the IC50 concentrations following a 48 hour treatment in MMQ cells (data not shown). These findings are entirely consistent with a previous study which reported a 2–3 fold greater immunosuppressive effect of rapamycin, as compared to RAD001.[19] Despite the differences in potency observed in cultured cells, it is notable that both rapamycin and RAD001 were equally effective *in vivo*.[23]

Rapamycin at a dose of 15 ng/mL (>15 nM serum concentration) is clinically well tolerated as an immunosuppressive agent to present transplant rejection;[4] however, a reduction in immune surveillance is likely undesirable in the context of tumor biology. Thus, it is noteworthy that picomolar concentration of rapamycin, a concentration that is <5% of the immunosuppressive concentration, effectively attenuated the growth of pituitary adenoma cells. Similarly, the concentration of RAD001 required for growth suppression in GH3 pituitary adenoma cells (986 pM) is clinically achievable, as a recently conducted Phase I clinical trial indicated that oral administration of 5 and 10 mg RAD001/daily was well tolerated and produced steady-state blood concentrations of 5.6 and 13.7 nM, respectively.[15] Although further testing in pre-clinical models remains to be performed, these findings suggest, for the first time, that rapamycin and FDA-approved rapamycin analogues such as RAD001 may represent a novel therapy to limit pituitary tumor growth.

Radiation remains a frontline and generally effective treatment option for pituitary adenoma patients; however, radiotherapy may be associated with hypopituitarism in ~33% of patients at 2 years and close to 100% of patients at 10 years. Similarly, radiosurgery is limited by the high sensitivity of surrounding structures, such as the optic apparatus, to radiation injury.[18] Thus, lowering the threshold for radiation-induced cell death may reduce the occurrence of detrimental side effects and improve long-term patient outcome. In support of a possible adjunct use for mTOR inhibitors in pituitary adenomas, clinically achievable concentrations of rapamycin and RAD001 (500 pM) enhanced the effect of low-dose radiation (2.5 Gy) on GH3 cells, which are generally considered to be radi-oresistant.[12,18] Importantly, the combination of either mTOR inhibitor with 2.5 Gy was more effective than either 2.5 Gy or 10 Gy (a radiation dose associated with damage to the optic apparatus) alone, suggesting that rapamycin and rapamycin derivatives may be clinically useful as an adjunct therapy to standard radiation treatment in pituitary tumors.

As a whole, these data suggest a possible use for mTOR inhibitors, either as a single agent to limit tumor growth and/or as an adjunct to radiation therapy. These findings therefore warrant further investigation in a pre-clinical animal model of pituitary tumors.
